# Supplementary material for: Disc Large Homolog 1 Is Critical for Early T Cell Receptor Micro Cluster Formation and Activation in Human T Cells
Source: Vaccines (Basel). 2021 Dec 7;9(12):1446. doi: 10.3390/vaccines9121446 (PMC8709471; doi:10.3390/vaccines9121446)
Supplement: Supplementary file 1 [file vaccines-09-01446-s001.zip › vaccines-1447895-supplementary.pdf]

Supplementary Figure S1 Dlg1 is critical for T cell receptor microcluster (TCR-MC) formation.

A and B. Microclusters of ZAP-70–YFP (green) and phosphorylated Y323 p38 (red) were imaged in immunostained Jurkat cells stably expressing ZAP-70–YFP. Cells were pretreated with control or DLG1 specific siRNA, then dropped onto coverslips coated with anti-CD3 (stimulated) or anti-CD45 (unstimulated) and visualized after 5 min. Scale bars, 10 µm. Data are representative of three experiments

C and D. ZAP70-YFP and P-Y323 p38 intensities were quantified using Image J in immunostained Jurkat cells stably expressing ZAP-70–YFP. Cells were pretreated with control or DLG1-specific siRNA, then dropped onto coverslips coated with anti-CD3 (stimulated) or anti-CD45 (unstimulated). Data are representative of 8 fields from 3 independent experiments.

E. Intracellular IL-2 production in human CD4+ and CD8+ T cells upon anti-CD3 +anti-CD28 stimulation for 18 hours.

Supplementary Figure S2 Sequence analysis of DLG1 KO clones

A. IGV snapshot of DLG1 KO clones

B. Allele analysis of Clonal populations

Supplementary Figure S3 Characteristic analysis of DLG1 KO cells

A. Immunoblot of DLG1 from wildtype and DLG1 KO Jurkat cell

B. TCR-ζ expression in wildtype and DLG1 KO Jurkat cell

C. Microcluster of phosphorylated Y493 ZAP-70 (red) and expression of DLG1 (blue) In immunostained wildtype or DLG1 KO Jurkat cells. Cells were dropped onto coverslips coated with anti-CD3 (stimulated) and visualized 5 min post stimulation.

Supplementary Figure S4 SHP2 knockdown fails to rescue the inhibition of T cell signaling conferred by DLG1 deficiency

Immunoblot analysis of lysates from wildtype and DLG1 KO Jurkat cells. Cells were pretreated with control or SHP2 specific siRNA, then activated with soluble anti-CD3 (monoclonal antibody OKT3) and antibody to mouse immunoglobulin G (IgG), followed by probing for phosphorylated and total proteins.

Figure S1

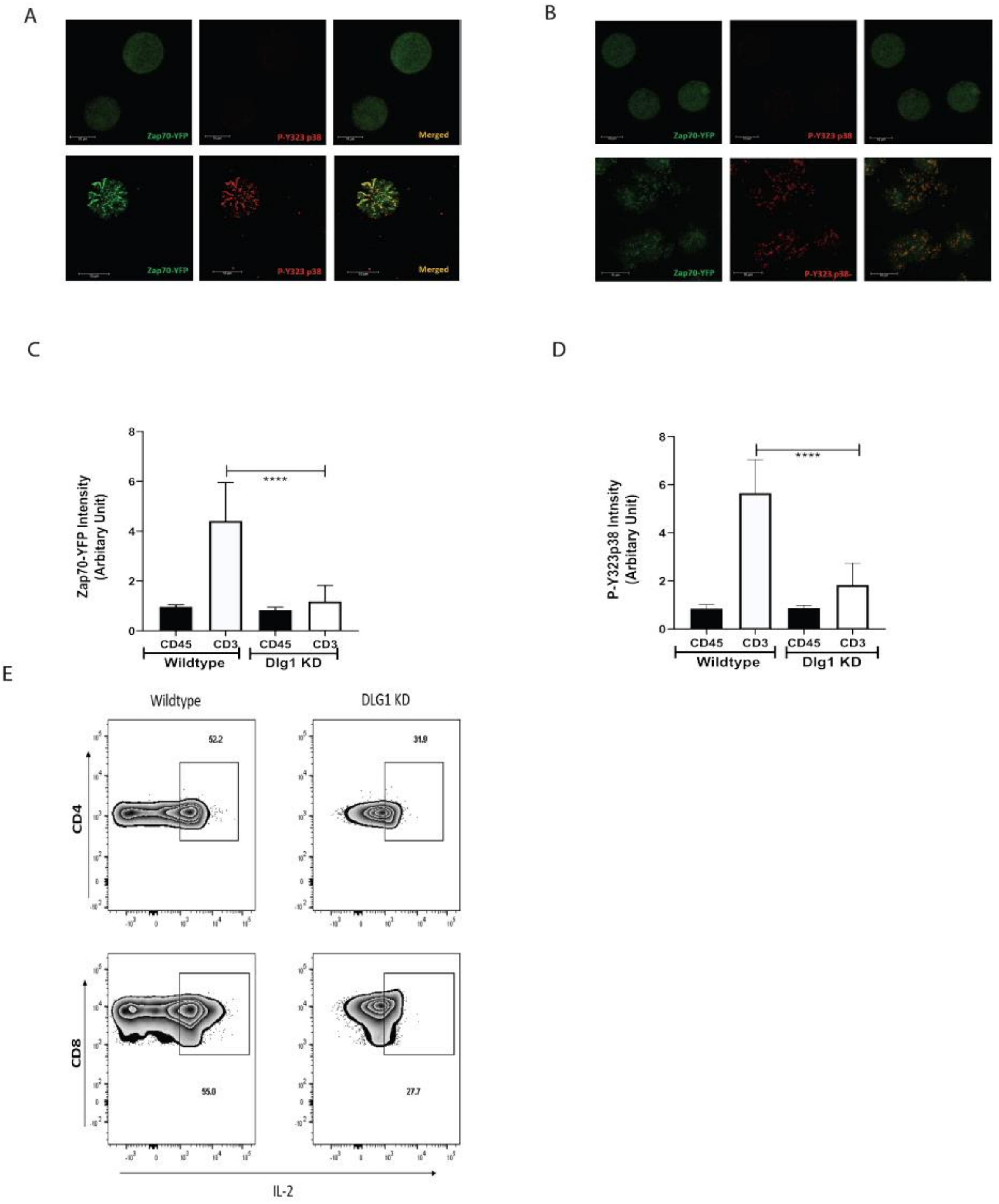
A
Zap70-YFP
P-Y323 p38
Merged
B
Zap70-YFP
P-Y323 p38
C
Zap70-YFP Intensity (Arbitary Unit)
****
CD45
CD3
CD45
CD3
Wildtype
Dlg1 KD
D
P-Y323p38 Intnsity (Arbitary Unit)
****
CD45
CD3
CD45
CD3
Wildtype
Dlg1 KD
E
Wildtype
DLG1 KD
52.2
31.9
CD4
CD8
55.0
27.7
IL-2

Figure S2

A

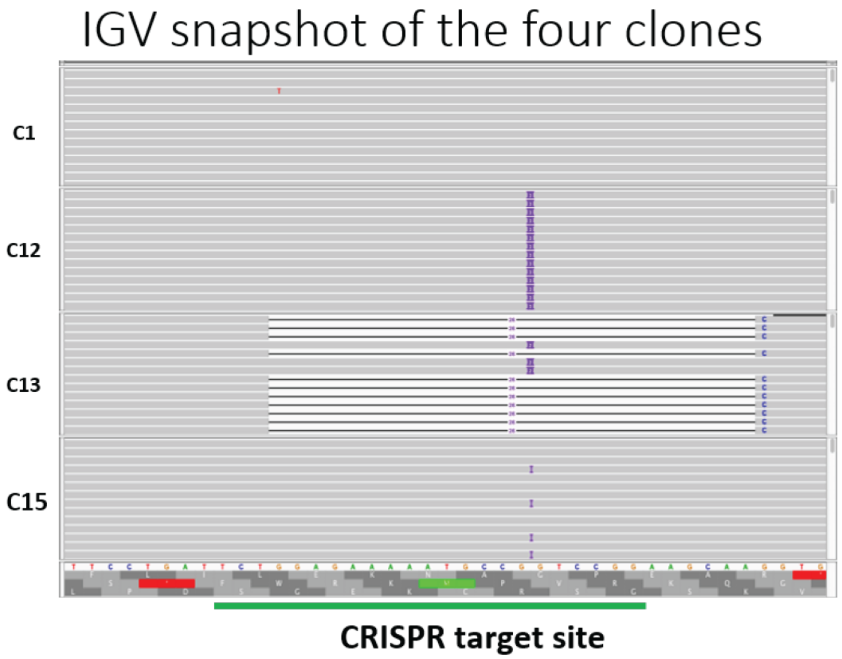

B

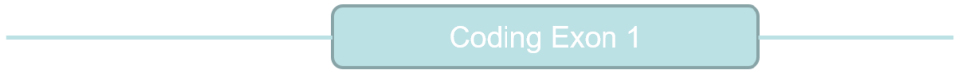

Clone TCTTCCTGAT<u>TCTGGAGAAAA**ATG**CCGGTCCGG</u>AAGCAAGGTGAGAGTTTATTCCGTAACT

C1 **Allele 1** TCTTCCTGATTCTGGAGAAAAATGCCGGTCCGGAAGCAAGGTGAGAGTTTATTCCGTAACT

Allele 2 TCTTCCTGATTCTGGAGAAAAATGCCGGTCCGGAAGCAAGGTGAGAGTTTATTCCGTAACT

C13 **Allele 1** TCTTCCTGATTCT---**C**GTGAGAGTTTATTCCGTAACT

Allele 2 TCTTCCTGATTCTGGAGAAAAATGCCG**TC**GTCCGGAAGCAAGGTGAGAGTTTATTCCGTAACT

C12 **Allele 1** TCTTCCTGATTCTGGAGAAAAATGCCG**CTC**GTCCGGAAGCAAGGTGAGAGTTTATTCCGTAACT

Allele 2 TCTTCCTGATTCTGGAGAAAAATGCCG**CTC**GTCCGGAAGCAAGGTGAGAGTTTATTCCGTAACT

C15 **Allele 1** TCTTCCTGATTCTGGAGAAAAATGCCG**T**GTCCGGAAGCAAGGTGAGAGTTTATTCCGTAACT

Allele 2 TCTTCCTGATTCTGGAGAAAAATGCCGGTCCGGAAGCAAGGTGAGAGTTTATTCCGTAACT

sgRNA target site underlined

Figure S3

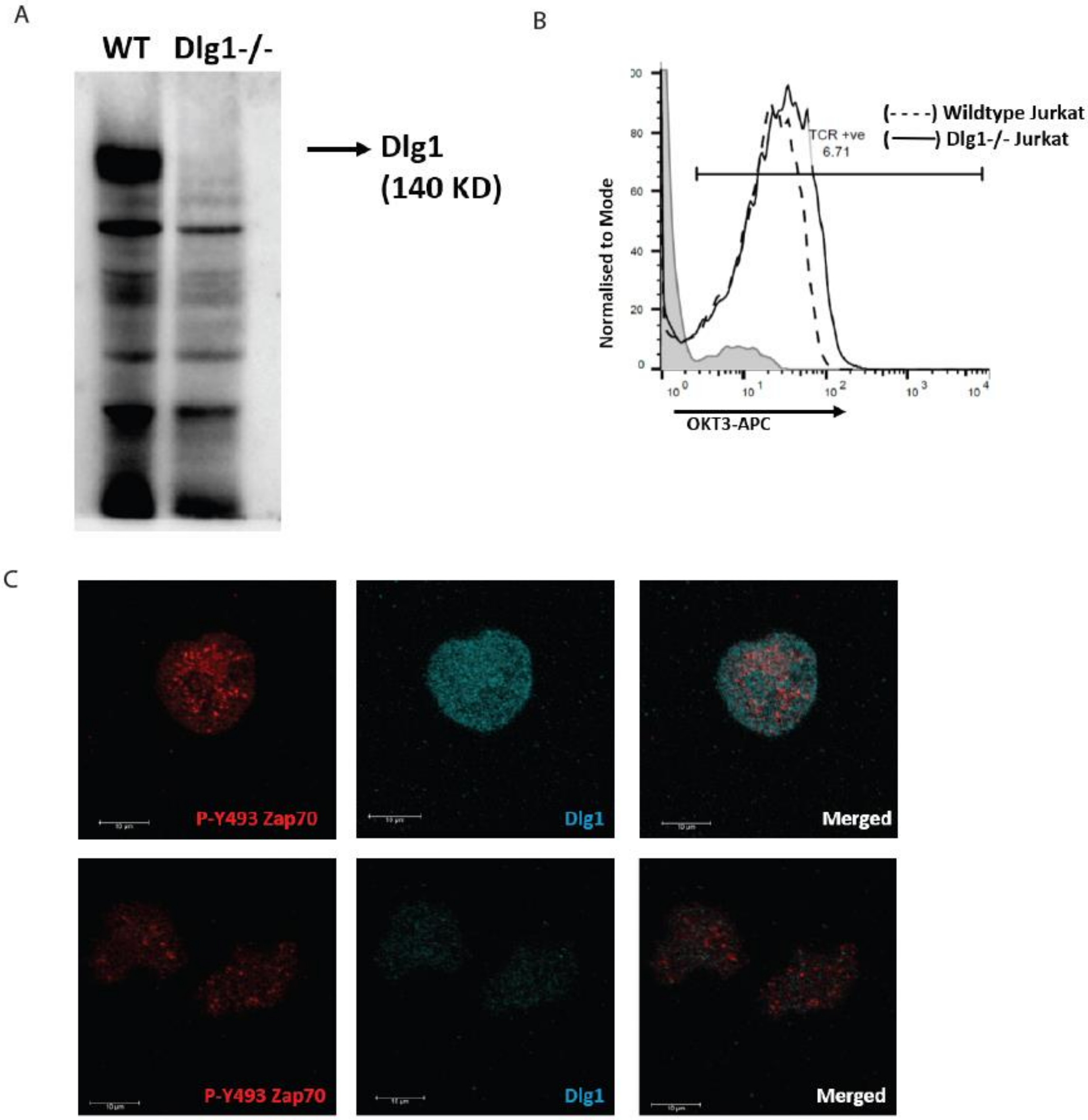
A
WT
Dlg1-/-
Dlg1
(140 KD)
B
Normalised to Mode
OKT3-APC
TCR +ve
6.71
(- - - -) Wildtype Jurkat
(——) Dlg1-/- Jurkat
C
P-Y493 Zap70
Dlg1
Merged
P-Y493 Zap70
Dlg1
Merged

Figure S4

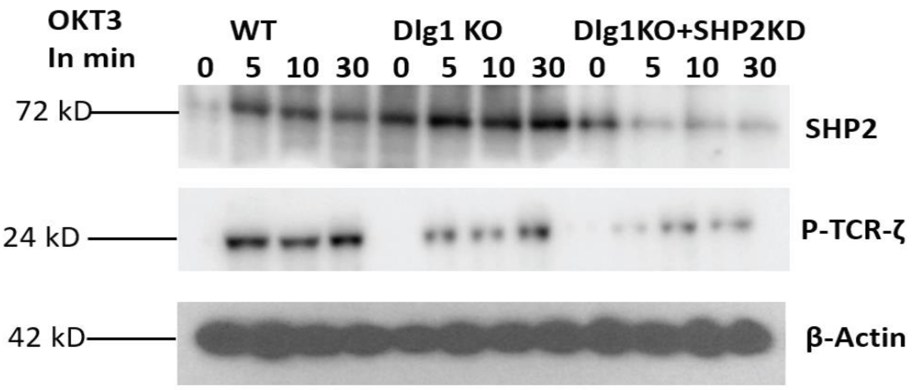
OKT3
In min
WT
Dlg1 KO
Dlg1KO+SHP2KD
0 5 10 30 0 5 10 30 0 5 10 30
72 kD
SHP2
24 kD
P-TCR-ζ
42 kD
β-Actin
